# Numerical simulation data on landslide generated impulse waves affected by the reservoir geometry

**DOI:** 10.1016/j.dib.2019.104938

**Published:** 2019-12-14

**Authors:** Yu Zhang, Deying Li, Lixia Chen, Kunlong Yin, Lili Xiao, Xiaolin Fu, Thomas Glade, Chin Leo

**Affiliations:** a Faculty of Engineering, China University of Geosciences, Wuhan 430074, China; b Institute of Geophysics and Geomatics, China University of Geosciences, Wuhan 430074, China; c School of Highway, Chang'an University, Xi'an 710064, China; d Wuhan Centre of China Geological Survey, Wuhan 430074, China; e Department of Geography and Regional Research, University of Vienna, Vienna 1010, Austria; f School of Computing, Engineering and Math, Western Sydney University, Sydney, NSW 1797, Australia

**Keywords:** Landslides, Impulse wave, Wave amplitude, Reservoir geometry

## Abstract

The data article provided time series of water surface elevation and wave parameters of landslide generated impulse waves affected by reservoir geometry. Two types of generalized reservoir geometries were investigated by the numerical method of Tsunami Squares–converging reservoir geometries and diverging reservoir geometries. 14 numerical reservoir models with expansion angle of 0°, 5°, 10°, 15°, 20°, 25° and 30° were performed. Time series of water surface elevation at the propagation distance of 2 km for the converging and diverging reservoir models were obtained from 70 numerical wave gauges, which allows to analyse the effect of reservoir geometries on wave characteristics generated by landslide. The wave profiles of section C at different propagation distance were also investigated in order to improve the understanding of wave characteristics at different positions. Furthermore, wave parameters (wave height, wave amplitude and wave trough) of different propagation distance for all reservoir geometries were present in the article. These data were used in Zhang et al., 2019 [1].

**Table:** Specifications Table

Subject	Earth and Planetary Sciences
Specific subject area	Engineering Geology/landslide generated impulse waves
Type of data	Text file (Excel data)
How data were acquired	Date set was collected and created by the numerical method of Tsunami Squares.
Data format	Raw
Parameters for data collection	The data were obtained from the numerical simulation of the diverging reservoir models and converging reservoir models with expansion angles of 0°, 5°, 10°, 15°, 20°, 25° and 30°. The 14 numerical experiments were performed using Tsunami Squares.
Description of data collection	Time series of water surface elevation at the fix distance, wave profiles and wave parameter (wave height, wave amplitude and wave trough) evolution at different distances for the diverging and converging reservoir geometries were investigated to analyse the wave decay characteristics and the effect of reservoir geometries on wave characteristics generated by landslide.
Data source location	The Three Gorges Reservoir in China
Data accessibility	Repository name: Mendeley Data Data identification number: https://doi.org/10.17632/mww43h5vs8.2 Direct URL to data: https://doi.org/10.17632/mww43h5vs8.2
Related research article	Author's name: Yu Zhang, Deying Li, Lixia Chen, Kunlong Yin, Lili Xiao, Xiaolin Fu, Thomas Glade, Chin Leo Title: Numerical analysis of landslide-generated impulse waves affected by the reservoir geometry. Journal: Engineering Geology DOI:https://doi.org/10.1016/j.enggeo.2019.105390

**Table:** 

**Value of the Data** • The data was collected from 14 numerical experiments of investigating the effect of reservoir geometries on landslide generated impulse waves, that can be used by other researchers as benchmark data set in the flied of landslide induced impulse waves. • Data sets can be used to investigate the wave characteristics of landslide induced impulse waves and wave parameters evolution with propagation distances. The data might be beneficial for hazard assessment to landslides with potential waves. • For future investigation on the effects of reservoir geometry on wave parameters, the data set can be further extended to enhance the analysis of wave characteristics of different reservoir models.

## 1 Data

The data shows the diverging reservoir models and converging reservoir models with expansion angles of 0°, 5°, 10°, 15°, 20°, 25° and 30°. Diverging reservoir model represents that impulse waves travelled from a narrow reservoir section to a wide one. The model dimensions were 6600 m (length) × 500 m (width in the middle) × 100 m (water depth). The landslide was located in the middle and the distance between the landslide source and the position where the channel became narrower was 1000 m. The model was symmetrical. The expansion angle with θ = 0, 5, 10, 15, 20, 25, and 30° were investigated in the diverging reservoir models. The converging reservoir model represents that landslide induced impulse waves propagated from a wide reservoir section into a narrower section. The model dimensions were 6600 m (length) × 3000 m (width in the middle) × 100 m (water depth). It was also symmetrical.

We set up the five sections (A, B, C, D and E) in the numerical models. The section C was located in the center of the reservoir. The distance of section B or section D was equal to a water depth away from the reservoir shore. For section A or section E, the distance away from the shore was half of a water depth. The slope angle of landslide source area was set up to 45°. The height of the landslide crown was 340 m above the water level of 150 m. The still water depth was 100 m and it was 50 m for sections A and E. The numerical wave gauges were set up in the converging reservoir model and diverging reservoir model. The distance between the monitoring points of the sections were set up to 200 m [1].

The data set is hosted in a public repository of Mendeley Data. The direct URL to data is https://doi.org/10.17632/mww43h5vs8.2. The five excel files were stored in it. It includes the water surface elevation of five sections at the distance of 2.0 km for the diverging reservoir models (file 1) and converging reservoir models (file 2), time series of water surface elevation of section C at different propagation distances of 0.8, 1.0, 1.4 and 1.8 km for the diverging reservoir geometries (file 3), time series of water surface elevation of section C at different distances of 1.0, 1.2, 1.4, 1.6, 1.8 and 2.0 km for the 0°, 15° and 30° converging reservoir geometries (file 4), wave parameters (wave height, wave amplitude and wave trough) with different propagation distance at section C in the diverging reservoir models (file 5) and converging reservoir models (file 6).

## 2 Experimental design, materials, and methods

To explore the effect of reservoir geometry on the propagation of landslide generated impulse waves in the TGR, two types of generalized reservoir geometries were established based on the characteristics of reservoir geometries: converging reservoir model and diverging reservoir model. Diverging expansion angle expressing the variation in reservoir geometries were investigated and the expansion angles were respectively set up to 0,5,10,15,20,25,and30° for the converging and diverging models. 14 numerical reservoir models were established.

These reservoir models were 6.6 km long and the still water depth was 100 m. At the location of landslide source, the convergence reservoir model was 3000 m width, while the divergence model was 500 m width. The distance away from landslide source to the starting changing in the reservoir width at different expansion angles was 1000 m. The slope angle of reservoir shore was 45°. Five monitoring sections (A, B, C, D and E) were set up in the reservoir models. The numerical wave gauges were installed at spacing of 200 m to record water surface elevation in the whole progress. The Gongjiafang landslide in the Three Gorges Reservoir, China was chosen as an identical wave generator for landslide generated impulse waves in the fourteen geometries. As the reservoir model was symmetrical, the landslide was set up to a symmetrical one through modifying landslide morphology on condition of keeping landslide volume constant.

The numerical method called Tsunami Squares *(TS)* was chosen to simulate landslide generated impulse wave propagation in the study [2,3]. Wave profiles at the distance of 2 km were obtained from 70 numerical wave gauges to investigate the effect of reservoir geometry on landslide induced impulse wave. The wave height, wave amplitude and wave trough at the section C were chosen as main wave parameters to investigate wave propagation characteristics of all the reservoir geometries including the converging reservoir model and diverging reservoir model. The wave profiles of section C at different distances of 0.8, 1.0, 1.4 and 1.8 km were investigated in order to improve the understanding of wave characteristics at different positions. The water surface elevation over time for all diverging reservoir geometries was recorded. In order to explore the wave characteristics at different distances of 1.0, 1.2, 1.4, 1.6, 1.8 and 2.0 km, the wave profiles of section C were also recorded for the converging reservoir geometries with the expansion angles of 0°, 15° and 30°.
